# Serum Levels of Fibroblast Growth Factor 19 Are Inversely Associated with Coronary Artery Disease in Chinese Individuals

**DOI:** 10.1371/journal.pone.0072345

**Published:** 2013-08-07

**Authors:** Yaping Hao, Jian Zhou, Mi Zhou, Xiaojing Ma, Zhigang Lu, Meifang Gao, Xiaoping Pan, Junling Tang, Yuqian Bao, Weiping Jia

**Affiliations:** 1 Department of Endocrinology and Metabolism, Shanghai Jiao Tong University Affiliated Sixth People’s Hospital, Shanghai Clinical Center for Diabetes, Shanghai Key Clinical Center for Metabolic Disease, Shanghai Diabetes Institute, Shanghai Key Laboratory of Diabetes Mellitus, Shanghai, China; 2 Department of Cardiology, Shanghai Jiao Tong University Affiliated Sixth People’s Hospital, Shanghai, China; College of Pharmacy, University of Florida, United States of America

## Abstract

**Background:**

The fibroblast growth factor 19 (FGF19) has been implicated in recent studies as a potential regulator of glucose and lipid metabolism, which may lead to atherosclerosis. Here, we investigated the association of FGF19 with the presence and severity of coronary artery disease (CAD) in a Chinese population.

**Methods:**

A total of 315 patients with suspected or established CAD, including 205 males and 110 postmenopausal females, were enrolled and assessed by coronary angiography. CAD severity was determined by the Gensini score. Serum FGF19 was measured by quantitative sandwich ELISA.

**Results:**

FGF19 levels were not significantly different between male and female patients (median [interquartile range], 143.40 [87.96–250.80] vs. 141.60 [87.13–226.32] pg/mL, *P* = 0.773). CAD patients had lower levels of FGF19 than those without CAD (128.20 [80.62–226.58] vs. 188.00 [105.10–284.70] pg/mL, *P* = 0.007). FGF19 was negatively correlated with 2hPG (*r* = –0.150, *P* = 0.008), FINS (*r* = –0.169, *P* = 0.004), HOMA-IR (*r* = –0.171, *P* = 0.004), and the Gensini score (*r* = –0.141, *P* = 0.012), but positively correlated with HDL-c (*r* = 0.116, *P* = 0.041) and adiponectin (*r* = 0.128, *P* = 0.024). Moreover, FGF19 was found to be independently correlated with 2hPG (*β* = –0.146, *P* = 0.022) and adiponectin (*β* = 0.154, *P* = 0.016). After adjusting for other CAD risk factors, FGF19 was demonstrated to be an independent factor for Gensini score (*β* = –0.140, *P* = 0.019) and the presence of CAD (*β* = –1.248, *P* = 0.036).

**Conclusions:**

Serum FGF19 is associated with the presence and severity of CAD in a Chinese population.

## Introduction

Fibroblast growth factor 19 (FGF19) is mainly synthesized by the intestinal epithelium, and is believed to function predominantly as a regulator of human bile acid metabolism [1], [2]. Recent studies, however, have uncovered a role of FGF19 in maintaining the balance of energy metabolism, presumably through its functions as an endocrine hormone [3], [4]. Indeed, when FGF19 was overexpressed in a transgenic mouse model, the major phenotypic effects were lower levels of body weight and cholesterol than the wild-type mouse, and it was determined that FGF19 played a vital role in maintaining normal glucose tolerance and insulin sensitivity [5]. Similar results were obtained when high-fat-fed mice were treated with recombinant FGF19 [6].

Despite these findings and the well-established link between metabolic disorders and cardiovascular disease, few clinical studies have reported on potential association of FGF19 with coronary artery disease (CAD). A decrease in FGF19 levels has been demonstrated in patients with metabolic syndrome (MS), obesity, and non-alcoholic fatty liver disease [7]–[9]. Each of these three particular diseases is theorized to be predisposing factor of CAD, and the relation with FGF19 may suggest its contribution to CAD.

The current study was designed to investigate the relationship between FGF19 and CAD in human patients, using coronary angiographic findings (the “gold standard” of CAD diagnosis) and the Gensini score (to assess the severity of CAD [10]) to perform a correlation analysis of the factor-disease interrelation, including relations with various demographic and clinical factors.

## Methods

### Ethics Statement

The Ethics Committee of Shanghai Jiao Tong University Affiliated Sixth People’s Hospital approved our study. All study participants provided written informed consent.

### Study participants

Patients presenting at the Department of Cardiology of Shanghai Jiao Tong University Affiliated Sixth People’s Hospital between July 2008 and Jan 2010 with suspected or established CAD were recruited for the study. Each study participant underwent coronary angiography and completed a standardized questionnaire about previous and present illness, medication history, and smoking status. Patients with the following characteristics were excluded from study enrollment: severe liver and kidney disease; biliary system disorders; recent myocardial infarction (<3 months prior); acute coronary syndrome; symptomatic heart failure; major trauma or surgery; or presence of tumor. A total of 315 patients, including 205 males and 110 postmenopausal females (mean age: 66.4±10.1 years), were enrolled for analysis.

### Diagnostic criteria

Coronary angiographic examinations were performed using the standard Judkins techniques [11], and angiograms were assessed by two experienced cardiologists who were blinded to the clinical traits of the patients. CAD diagnosis was made according to the presence of ≥50% stenosis in ≥1 main coronary artery. The Gensini score system was used to quantitatively assess the extent of each observed coronary lesion.

### Anthropometric evaluation

Weight and height were recorded and used to calculate the body mass index (BMI; kg/m^2^). Waist circumference (W) was measured at the midpoint between the inferior border of the lowest rib and the upper margin of the iliac crest on the midaxillary line.

### Biochemical measurements

Patients were instructed to fast overnight (10 h), and venous blood samples were collected and stored at –80°C until use. The Hitachi 7600-020 auto-analyzer (Tokyo, Japan) was used to measure fasting plasma glucose (FPG), 2 h postprandial glucose (2hPG) (both by the glucose oxidase method), and lipid profiles, including total cholesterol (TC), triglyceride (TG), low-density lipoprotein cholesterol (LDL-c) and high-density lipoprotein cholesterol (HDL-c) (by enzymatic methods). The Bio-Rad Variant II high-pressure liquid chromatogram (Hercules, CA, USA) was used to measure glycated hemoglobin A1c (HbA1c). Radioimmunoassay was used to measure serum fasting insulin (FINS) (Linco Research Inc., St Charles, MO, USA). The homeostasis model assessment index (HOMA-IR) was used to assay insulin resistance (IR) [12]. Quantitative sandwich enzyme-linked immunosorbent assay (ELISA) kits (from Li Ka Shing Faculty of Medicine, The University of Hong Kong) were used to measure serum FGF19 (with intra- and inter-assay coefficients of variation of 3.91% and 7.07%, respectively) and adiponectin (with intra- and inter-assay coefficients of variation of 7.34% and 8.64%, respectively). A particle-enhanced immunonephelometry analyzer (Dade-Behring Inc., Newark, NJ, USA) was used to assess C-reactive protein concentration.

### Statistical analysis

All statistical analyses were carried out with the SPSS 16.0 statistical software package (SPSS Inc., Chicago, IL, USA). The one-sample Kolmogorov-Smirnov test was performed to determine normality of the data distribution. Normally distributed data were expressed as mean ± standard deviation (SD), and data with skewed distribution were expressed as median with interquartile range. Comparison of continuous data between the two groups was carried out by using an unpaired Student’s *t*-test (normal distribution) or the Mann-Whitney U-test (skewed distribution), and of categorical variables by using the Chi-squared (χ^2^) test. Spearman’s correlation coefficient analysis was conducted to assess the relationships between FGF19 and other metabolic parameters. Multiple stepwise regression analysis was used to explore the influence of different variables on FGF19 and to adjust for covariates. Independent factors were sex, age and all the metabolic-related variables including BMI, W, blood pressure, FPG and 2hPG, lipid profiles, HOMA-IR, adiponectin and CRP along with disease-related therapies such as statin use, anti-hypertensive therapy and anti-diabetic therapy. To determine the independent predictors for the presence and severity of CAD, all the conventional risk factors related with CAD as well as the disease-related therapies were tested in multivariate logistic regression and multiple stepwise regression analysis respectively. The threshold of statistical significance was set at 0.05 for two-tailed *P*-values.

## Results

Among the total 315 study participants, serum FGF19 levels ranged from 18.66 to 607.60 pg/mL. Compared with men, women showed significantly higher age, TC, HDL-c, LDL-c, adiponectin, and lower levels of W and 2hPG (*P = *0.001∼0.027). Moreover, women had significantly lower proportions of smoking and CAD (*P* = 0.001 and *P*<0.001 respectively). FGF19 levels were not significantly different between the two groups (143.40 [87.96-250.80] vs. 141.60 [87.13-226.32] pg/mL, *P = *0.773). Additionally, there were no significant differences in BMI, systolic or diastolic blood pressure (SBP or DBP), FPG, HbA1c, TG, FINS, HOMA-IR, or CRP between men and women (*P*>0.05); in addition, the two groups had similar proportions of use of therapeutic statins, anti-hypertensives, and anti-diabetic drugs.

**Figure 1:**
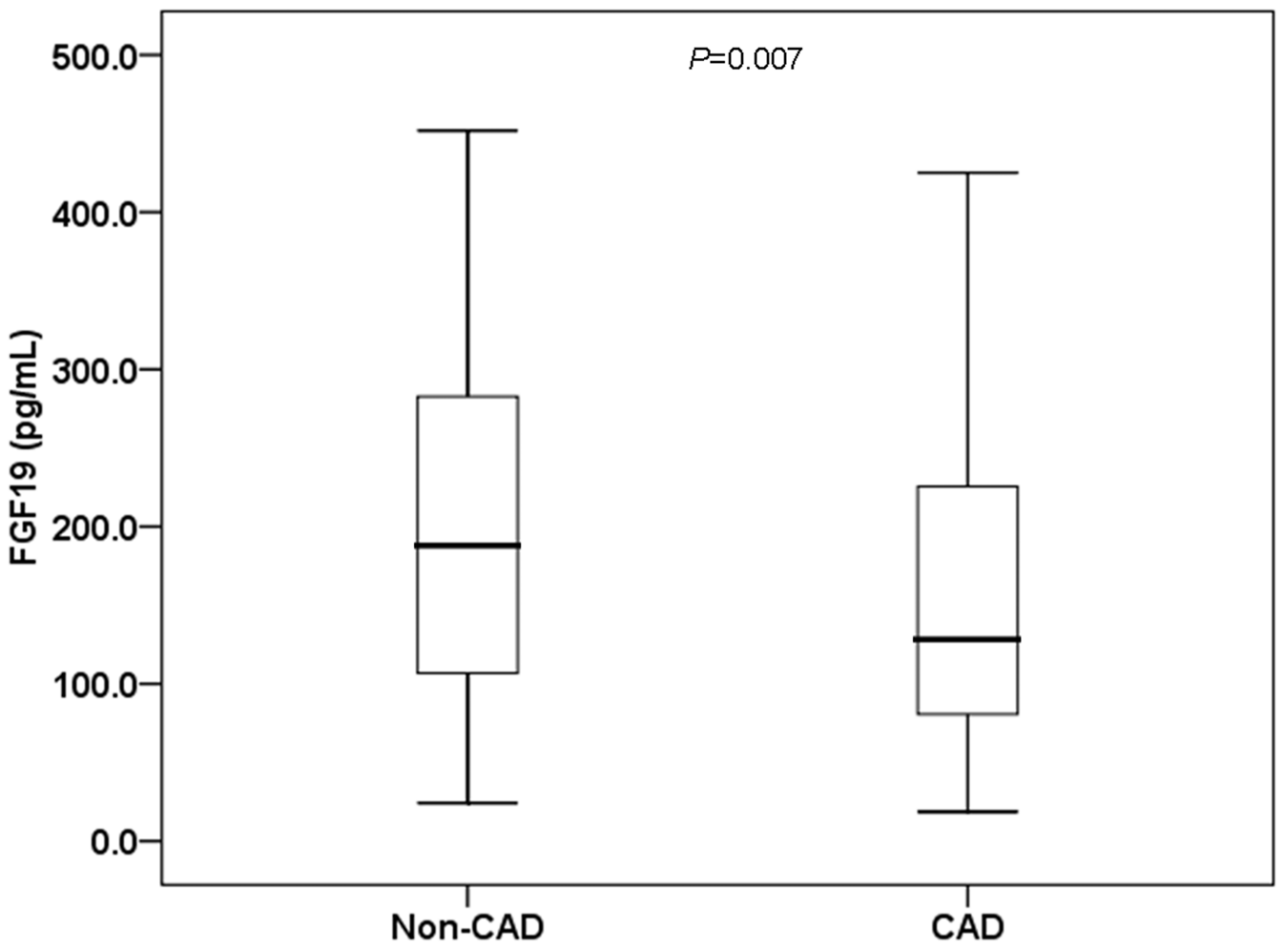
Comparison of serum FGF19 levels in the non-CAD and CAD groups. The FGF19 levels were 188.00 (105.10-284.70) pg/mL in the non-CAD group and 128.20 (80.62-226.58) pg/mL in the CAD group.

Coronary angiography revealed CAD in 228 of the study participants (Table 1). The CAD patients were characterized as predominately male, and had significantly higher age, 2hPG, proportion of therapeutic statins use and anti-diabetic drugs, but significantly lower levels of TC and HDL-c (*P* = 0.001∼0.045). In addition, the CAD patients showed significantly lower levels of FGF19 (CAD: 128.20[80.62-226.58] vs. non-CAD: 188.00[105.10-284.70] pg/mL, *P* = 0.007; Fig. 1).

**Table 1:** Demographic and clinical characteristics of study participants.

Variables	Total	Non-CAD	CAD	P
Men/women	205/110	44/43	161/67	0.001
Age (year)	66.39 ± 10.00	64.51 ± 9.69	67.11 ± 10.04	0.038
BMI (kg/m^2^)	24.53 ± 3.24	24.83 ± 3.74	24.41 ± 3.02	0.312
W (cm)	90.16 ± 9.64	90.25 ± 11.15	90.13 ± 9.03	0.917
SBP (mmHg)	130 (120–148)	130 (120–148)	130 (120–149)	0.492
DBP (mmHg)	80 (70–84)	80 (70–85)	80 (70–80)	0.397
FPG (mmol/L)	5.44 (5.04–6.29)	5.45 (5.07–6.11)	5.44 (5.02–6.39)	0.822
2hPG (mmol/L)	8.55 (6.60–11.59)	8.18 (6.23–9.83)	8.75 (6.69–11.96)	0.045
HbA1c (%)	6.2 (5.8–6.7)	6.1 (5.7–6.5)	6.2 (5.8–6.9)	0.107
TC (mmol/L)	4.32 ± 1.01	4.52 ± 0.98	4.23 ± 1.01	0.025
TG (mmol/L)	1.49 (1.07–2.13)	1.56 (1.01–2.24)	1.48 (1.07–2.07)	0.691
HDL-c (mmol/L)	1.06 (0.88–1.31)	1.10 (0.90–1.41)	1.01 (0.87–1.27)	0.031
LDL-c (mmol/L)	2.94 ± 0.93	3.06 ± 0.85	2.90 ± 0.96	0.163
FINS (mU/L)	15.96 (11.48–21.60)	16.54 (12.41–21.67)	15.78 (10.99–21.43)	0.707
HOMA-IR	4.00 (2.78–5.85)	4.03 (2.87–5.87)	3.99 (2.64–5.80)	0.733
Adiponectin (mg/L)	7.48 (5.03–12.10)	8.40 (5.72–12.17)	7.16 (4.91–12.03)	0.220
CRP (mg/L)	1.25 (0.55–3.21)	1.46 (0.54–4.00)	1.19 (0.54–3.17)	0.836
Smoking, N (%)	138 (43.8)	32 (36.8)	106 (46.5)	0.129
Statins therapy, N (%)	87 (27.6)	12 (13.8)	75 (32.9)	0.001
Anti-hypertensives, N (%)	217 (68.9)	60 (69.0)	157 (68.9)	1.000
Anti-diabetic drugs, N (%)	73 (23.2)	13 (14.9)	60 (26.3)	0.036

Abbreviations: BMI, body mass index; W, waist circumference; SBP, systolic blood pressure; DBP, diastolic blood pressure; FPG, fasting plasma glucose; 2hPG, 2-h postchallenge glycemia; HbA1c, glycated hemoglobin A1c; TC, total cholesterol; TG, triglyceride; HDL-c, high density lipoprotein cholesterol; LDL-c, low density lipoprotein cholesterol; HOMA-IR, homeostasis model assessment index of insulin resistance; CRP, C-reactive protein. Data are means ± SD or median (interquartile range).

We performed a correlation analysis between anthropometric, biochemical variables and FGF19. As expected, FGF19 levels were found to be negatively correlated with 2hPG (*r* = –0.150, *P* = 0.008), FINS (*r* = –0.169, *P* = 0.004), HOMA-IR (*r* = –0.171, *P* = 0.004) and Gensini score (*r* = –0.141, *P* = 0.012) but positively correlated with HDL-c (*r* = 0.116, *P* = 0.041) and adiponectin (*r* = 0.128, *P* = 0.024) (Table 2). Multiple stepwise regression analysis using FGF19 as the dependent variable identified 2hPG (*β* = –0.146, *P* = 0.022) and adiponectin (*β* = 0.154, *P* = 0.016) as independent predictors after adjustments for sex, age, BMI, W, blood pressure, glucose, lipid profiles, HOMA-IR, adiponectin, CRP, and use of therapeutic statins, anti-hypertensives, and anti-diabetic drugs (Table 3).

**Table 2:** Correlation of FGF19 with anthropometric parameters and biochemical indexes.

Variables	FGF19	
	*r*	*P*
Age	0.002	0.976
BMI	–0.051	0.368
W	–0.086	0.130
SBP	0.057	0.316
DBP	0.104	0.068
FPG	–0.051	0.372
2hPG	–0.150	0.008
HbA1c	–0.099	0.084
TC	0.102	0.074
TG	0.064	0.265
HDL-c	0.116	0.041
LDL-c	0.109	0.055
FINS	–0.169	0.004
HOMA-IR	–0.171	0.004
Adiponectin	0.128	0.024
CRP	–0.012	0.834
Gensini score	–0.141	0.012

Abbreviation: FGF19, fibroblast growth factor 19; BMI, body mass index; W, waist circumference; SBP, systolic blood pressure; DBP, diastolic blood pressure; FPG, fasting plasma glucose; 2hPG, 2-h postchallenge glycemia; HbA1c, glycated hemoglobin A1c; TC, total cholesterol; TG, triglyceride; HDL-c, high density lipoprotein cholesterol; LDL-c, low density lipoprotein cholesterol; FINS, fasting insulin; HOMA-IR, homeostasis model assessment index of insulin resistance; CRP, C-reactive protein.

**Table 3:** Variables independently associated with FGF19, as identified by linear regression analysis*.

Variables	*β*	*SE*	*P*
2hPG	–0.146	0.005	0.022
Adiponectin	0.154	0.002	0.016

* Variables included in the original model were sex, age, BMI, W, SBP, DBP, FPG, 2hPG, TC, TG, HDL-c, LDL-c, HOMA-IR, CRP, adiponectin, therapeutic use of statins, anti-hypertensives, or anti-diabetic drugs.

Abbreviation: FGF19, fibroblast growth factor 19; 2hPG, 2-h postchallenge glycemia.

To determine which variables were independently associated with Gensini score, multiple stepwise regression analysis using the Gensini score as the dependent variable identified sex, age, BMI, W, blood pressure, FPG, 2hPG, lipid profiles, HOMA-IR, FGF19, adiponectin, CRP, smoking, use of therapeutic statins, anti-hypertensives, and anti-diabetic drugs as independent variables. After adjustments, only FGF19 (*β* = –0.140, *P* = 0.019), use of therapeutic statins (*β* = 0.278, *P*<0.001), and female sex (*β* = –0.256, *P*<0.001) retained significance for independently predicting the Gensini score (Table 4).

**Table 4:** Variables independently associated with Gensini score, as identified by linear regression analysis*.

Variables	*β*	*SE*	*P*
FGF19	–0.140	0.329	0.019
Statins therapy	0.278	0.209	<0.001
Female sex	–0.256	0.196	<0.001

* The dependent variable was Gensini score [Ln(Gensini score+1)]. Variables included in the original model were FGF19, sex, age, BMI, W, SBP, DBP, FPG, 2hPG, TC, TG, HDL-c, LDL-c, HOMA-IR, CRP, adiponectin, smoking, and therapeutic use of statins, anti-hypertensives, or anti-diabetic drugs.

Abbreviation: FGF19, fibroblast growth factor 19.

In order to assess which variables were independently associated with the presence of CAD, multivariate Logistic regression analysis using the presence of CAD as the dependent variable was performed. The independent variables included sex, age, BMI, W, blood pressure, FPG, 2hPG, lipid profiles, HOMA-IR, FGF19, adiponectin, CRP as well as smoking, statin use, anti-hypertensive therapy and anti-diabetic therapy. As a result, FGF19 (*β* = –1.248, *P* = 0.036), use of statin (*β* = 1.348, *P* = 0.002) and female gender (*β* = –1.320, *P* = 0.006) were independent predictors of the presence of CAD (Table 5).

**Table 5:** Multivariate logistic regression analysis showing factors independently associated with the presence of CAD*.

Independent Variable	*β*	*S.E.*	*P*	OR	*95%*CI
FGF19	–1.248	0.597	0.036	0.287	0.089–0.924
Statin	1.348	0.432	0.002	3.850	1.650–8.984
Female gender	–1.320	0.478	0.006	0.267	0.105–0.681

* Variables of the original model included: FGF19, sex, age, BMI, W, SBP, DBP, FPG, 2hPG, TC, TG, HDL-c, LDL-c, HOMA-IR, CRP, adiponectin, smoking, and therapeutic use of statins, anti-hypertensives, or anti-diabetic drugs.

Abbreviation: FGF19, fibroblast growth factor 19.

## Discussion

The association of FGF19 with various metabolic disorders which are recognized as promoting factors of atherosclerosis development is well-studied. To date, however, only one study explored the association of FGF19 with the plasma atherosclerosis index (AIP; defined as log[TG/HDL-c]) which was a simple indicator of CAD. Ultimately, the authors demonstrated a negative relationship between FGF19 and AIP (*r* = –0.312, *P* = 0.05) that existed in MS patients with type 2 diabetes [13]. In the current study, coronary angiography (considered as the “gold standard” for CAD diagnosis) and Gensini score were analyzed and it revealed that CAD presence and severity were associated with decreased FGF19 levels. Specifically, regression analysis demonstrated that FGF19 was independently associated with the Gensini score. To the best of our knowledge, this is the first study to show that serum FGF19 levels are associated with CAD, and justifies future studies to explore the mechanistic role of FGF19 in CAD pathogenesis.

The putative protective effect of FGF19 in relation to CAD might involve its influence on metabolic disorders. In addition to the negative correlation of FGF19 with MS and HbA1c (*r* = –0.357, *P* = 0.028) in Turkish patients reported by Barutcuoglu *et al*. [13], another study of Czech participants by Stejskal *et al*. demonstrated a negative correlation of FGF19 with glucose (*r* = –0.350, *P*<0.01) and a positive correlation with HDL-c (*r* = 0.24, *P* = 0.045) [7]. Similarly, in the present study of ethnic Chinese patients, FGF19 appeared to be closely associated with 2hPG and HDL-c levels. Moreover, the Chinese patients revealed a negative association between FGF19 and insulin resistance, which supported the previous findings [5].

The mechanisms underlying the observed interrelation between FGF19 and various metabolic-related factors and conditions have been explored in several studies. The data reported by Fu [6] *et al*. showing that treatment with FGF19 in brown adipose tissue (BAT) deficient or leptin depleted mice could benefit glucose and lipid profiles, indicated that FGF19 could improve metabolic disorders and insulin sensitivity via mechanisms which were independent of the leptin signaling pathway and the thermogenic process of BAT. Further studies of the mechanism by which FGF19 lowers lipid levels have indicated that its inhibition of acetyl CoA carboxylase 2 (ACC2) and stearoyl-coenzyme A desaturease-1 (SCD1) leads to increase in fatty acid oxidation [14]–[17], which improves metabolism.

As previously shown in a study of German patients on chronic hemodialysis [18], the current study in a Chinese cohort of CAD and non-CAD patients also demonstrated an independent relationship between FGF19 and adiponectin which was suggested to be beneficial to metabolic profile. Adiponectin is an anti-inflammatory factor highly expressed in adipose tissue and exerts its function on endothelial cells; as such, adiponectin plays vital roles in both physiological conditions, such as glucose and lipid metabolism, as well as pathogenic conditions, such as insulin resistance and arteriosclerosis [19]. Indeed, a protective effect against atherosclerosis was demonstrated in a mouse model of apolipoprotein-E deficiency upon overexpression of globular adiponectin [20]. Intriguingly, human CAD patients have decreased levels of adiponectin [21], and a 10-year prospective study of elderly men demonstrated that high baseline adiponectin levels were related with low risk of CAD [22]. The majority of patients in our study had a history of therapeutic statins use prior to enrollment. While it has been previously reported that statins can increase adiponectin levels in CAD patients [23], no direct association between adiponectin and CAD was observed in the present study. However, our study did find that use of therapeutic statins was positively correlated with CAD. This finding may simply reflect the biased composition of our study population towards individuals at high risk for CAD and/or the fact that statin use was higher in CAD patients than in non-CAD subjects. The last finding in our current study was that female sex appeared to be a protective factor against the severity of coronary lesions, as has been previously shown [24].

When interpreting our findings, however, certain limitations in the study design must be considered as they may have impacted the results. First, study participants were selected from a high-risk population (as stated above), and several had already received disease-related therapies (both for CAD and MS); future studies should seek to obtain a more heterogeneous population sample. Second, the sample size was relatively small, and restricted the inferences that can be made from the statistical analyses. Third, the current study was cross-sectional, which precluded our ability to determine the causal relationship between FGF19 and CAD.

## Conclusions

In conclusion, this study provides the first evidence of decreased serum FGF19 levels in CAD patients being correlated with the presence and severity of coronary lesions, suggesting that reduced FGF19 expression might be involved in the development and progression of CAD.
